# Electrocardiogram changes post surgical repair of tetralogy of Fallot with valve-sparing versus transannular patch: A retrospective observational study

**DOI:** 10.21542/gcsp.2024.28

**Published:** 2024-08-01

**Authors:** Hamood Al Kindi, Sultan Al Battashi, Moosa Al Lawati, Ismail Al Abri, Madan Mohan Maddali

**Affiliations:** 1 Division of Cardiothoracic Surgery, Department of Surgery, Sultan Qaboos University Hospital, Oman; 2 Department of Child Health, The Royal Hospital, Muscat, Oman; 3 National Heart Center, The Royal Hospital, Muscat, Oman

## Abstract

**Introduction:** Long-term survival after tetralogy of Fallot (TOF) repair depends on several factors including the extent of chronic right ventricular adaptation to surgery. QRS duration (QRSd) is an important determinant of life-threatening arrhythmia post-TOF repair. This single-center retrospective study was designed to evaluate changes in QRSd post-TOF repair using either a pulmonary valve-sparing approach (VSA) or transannular patch (TAP).

**Methods and results:** Data from patients who underwent TOF repair between January 2016 and December 2019 were analyzed to compare the changes in the QRSd following intracardiac repair after VSA (Group 1) or TAP (Group 2). Among the 105 patients who underwent TOF surgical repair, 60 were included in the study (Group 1:30 vs. Group 2:30). Electrocardiograms (ECGs) were recorded pre- and post-surgery. The primary outcome was to compare the change in QRSd (ΔQRSd) before and after surgery between the two groups. The mean length of postoperative follow-up was 35.9 months in group 1 and 34.47 months in group 2. The mean [SD] difference in QRSd values (QRSd2 − QRSd1) was shorter in Group 1 (45.67 [22.79] ms) than in Group 2 (49.63 [23.76] ms); however, these differences were not statistically significant (*p* = 0.428). The PR interval was similar between the two groups in both preoperative and postoperative ECG.

**Conclusion:** At the short-term follow-up, both surgical approaches (VSA and TAP) resulted in similar QRSd post-TOF repair. Studies with longer follow-up periods are required to evaluate the association of the surgical approach with prolongation of QRSd and mortality.

## Introduction

Tetralogy of Fallot (TOF) is the most common form of cyanotic congenital heart disease, where children are often at risk of ventricular arrhythmias and sudden cardiac death^1–4^. It is one of the first cardiac anomalies to be successfully repaired by congenital cardiac surgeons^1,2^. Obstruction of the right ventricular outflow tract (RVOT), ventricular septal defect (VSD), an overriding aorta, and hypertrophy of the right ventricle (RV) are the cardinal features associated with TOF^5^. Surgical intervention in TOF dates back to 1954, when techniques such as ventriculotomy were employed by placing a pulmonary transannular patch (TAP) to relieve the RVOT obstruction, or use of palliative care in the form of the Blalock–Taussig–Thomas shunt^2,6,7^. Currently, the strategies used in TOF treatment have witnessed a major paradigm shift, resulting in significant long-term survival (20-year survival of almost 60–83%), a remarkable pulmonary valve-sparing approach (VSA), and TAP repair^4,6,8,9^.

The pathophysiology of TOF manifests as a crosstalk between the anatomy of the heart, its electrical impulse, and its mechanical function^10,11^. To gain a proper insight into adverse patient outcomes, electrocardiogram (ECG) metrics of depolarization and repolarization or, more concisely, the QRS complex has always proven efficacious^12,13^. There is limited knowledge regarding the difference in ECG changes post-TOF repair based on the surgical approach. This study compares the ECG alterations post-TOF repair in pediatric patients from a single tertiary cardiac center involving the two important surgical approaches for TOF repair, VSA and TAP.

## Patients and methods

### Study population and design

This was a retrospective study of all infants with TOF who underwent surgical repair using either VAS or TAP at our institution. The surgical technique for the valve-sparing approach involved a combination of transatrial access and limited infundibular ventriculotomy with a patch on the right ventricular outflow tract. We included all children who had baseline preoperative ECG (ECG1) and late post-operative ECG (>1 year after surgery, ECG2). Children with TOF having pulmonary atresia, absence of pulmonary valve (PV), atrioventricular septal defect, and incomplete ECG data were all excluded from the study.

The primary outcome of this study was to compare the changes in QRSd following intracardiac repair using the VAS or TAP approach. The secondary outcomes were to compare the two techniques for the changes in the rhythm, PR intervals, and ECG findings of PV regurgitation, tricuspid valve regurgitation, and RV dilatation. Baseline characteristics, including sex, median age, associated syndromes, collaterals, side of the aortic arch, presence of cyanotic spells before surgery, and preoperative palliative procedures, were obtained for all patients. In addition, ECG data were recorded preoperatively (ECG1 for QRSd1) and at follow-up (ECG2 for QRSd2). Rhythm, heart rate, QRSd, and PR intervals were recorded from ECGs stored in the electronic hospital information system. To overcome the bias in the variation of the preoperative QRSd and the follow-up duration on the outcome, we matched both groups with a tolerance level of 5 ms in the preoperative ECG and 3 months of follow-up, respectively.

### Statistical analysis

Statistical Package for Scientific Studies (SPSS) version 13.0 for Windows (by IBM) was used for analysis. All parameters were analyzed descriptively. The Mann–Whitney test was used to evaluate differences between the two groups for continuous variables, and the Kruskal–Wallis test (non-parametric approach) was used to assess the differences between the two groups for categorical variables. Statistical differences were considered significant if the *p*-value was less than 0.05.

**Table 1:** Baseline characteristics of the patients enrolled in the study.

**Variables**	**Group 1** **VSA (*n* = 30)** ***n* (%)**	**Group 2** **TAP (*n* = 30)** ***n* (%)**	**Total** **(*n* = 60)** ***n* (%)**	***P***
**Nationality** Omani Non-Omani	29 (96.7) 1 (3.3)	28 (93.3) 2 (6.7)	57 (95.0) 3 (5.0)	1.000
**Gender** Male Female	17 (56.7) 13 (43.3)	19 (63.3) 11 (36.7)	36 (60.0) 24 (40.0)	0.792
**Age (months)** Mean [SD]	20 [17.0]	18 [26.0]	——	0.733
**Syndromic** Yes No	7 (23.3) 23 (76.7)	7 (23.3) 23 (76.7)	14 (23.3) 46 (76.7)	1.000
**MAPCAS** Yes No	3 (10.0) 27 (90.0)	4 (13.3) 26 (86.7)	7 (11.7) 53 (88.3)	1.000
**Aortic arch (L-R)** Yes No	22 (75.9) 7 (24.1)	25 (83.3) 5 (16.7)	47 (79.7) 12 (20.3)	0.532
**Palliative procedure** Yes No	6 (20.0) 24 (80.0)	1 (3.3) 29 (96.7)	7 (11.7) 53 (88.3)	0.103
**Preoperative cardiac catheterization procedures** Yes No	11 (36.7) 19 (63.3)	6 (20.0) 24 (80.0)	17 (28.3) 43 (71.7)	0.252

### Ethics

An institutional ethics review board was sought (SRC#104/2019) to obtain the required permission for the study.

**Table 2:** Change in QRSd (ΔQRSd) and the PR interval between the surgical approaches.

**Variables**	**Group 1** **VSA (*n* = 30)** **Mean (SD)**	**Group 2** **TAP (*n* = 30)** **Mean (SD)**	***P*** ^*^
Postoperative follow-up duration (months)	35.90 (13.11)	34.47 (13.29)	0.641
QRSd1 (preoperative) (ms)	67.27 (8.31)	70.70 (15.14)	0.583
QRSd2 (last follow-up) (ms)	112.93 (23.73)	120.33 (18.80)	0.370
QRSd2–QRSd1 (ΔQRSd) (ms)	45.67 (22.79)	49.63 (23.76)	0.428
PR1 (preoperative PR interval) (ms)	127 (18.0)	128 (26.0)	0.870
PR2 (last follow-up PR interval) (ms)	133 (24.0)	133 (21.0)	0.950

**Notes.**

* Mann–Whitney, Non-parametric test.

**Figure 1.**
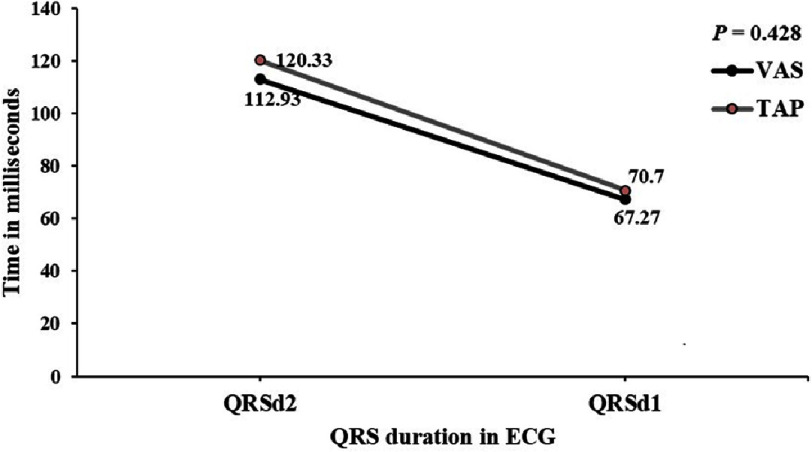
QRS duration in ECG of pediatric patients for VSA and TAP. The QRSd2 and QRSd1 were comparable between the two surgical interventions, with those for VSA being lesser, but not statistically significant.

## Results

### Patient baseline characteristics

Data from 105 pediatric patients who underwent surgical repair for TOF between January 2016 and December 2019 were available. In total, 79 patients had available ECGs before surgery (ECG1) and at the last follow-up visit (>1 year after surgery, ECG2). After matching the two groups based on postoperative follow-up duration, 60 patients were included in the study (Group 1 [VSA]: 30 *vs*. Group 2 [TAP]: 30). Baseline characteristics were balanced in both groups (Table 1). Approximately 60% of the patients were male and 23.3% were syndromic. The mean (SD) age of the pediatric patients was 20 (17.0) months and 18 (26.0) months for Groups 1 and 2, respectively. Most patients (79.7%) had an aortic arch (L-R) and 11.7% had major aortopulmonary collateral arteries (MAPCAS). Overall, 11.7% of patients underwent palliative procedures such as modified Blalock–Taussig shunt (MBTS), RVOT stent, patent ductus arteriosus (PDA) stent, or pulmonary valvuloplasty, and preoperative cardiac catheterization was offered to 28.3% of the patients.

### Change in QRSd and follow-up

The mean [SD] preoperative QRS duration (QRSd1) was similar between the two groups (Group 1:67.27 [8.31] ms *vs.* Group 2:70.70 [15.14] ms, *P* = 0.583) (Table 2). The mean postoperative follow-up duration was 35.9 months in Group 1 and 34.47 months in Group 2 (*P* = 0.641). At follow-up, the mean [SD] QRSd2 increased remarkably with time (post-surgery from baseline ECG) in both groups (Group 1:112.93 [23.73] ms *vs.* Group 2:120.33 [18.80] ms); however, the intergroup variation was not significant (*P* = 0.370). The mean [SD] difference between QRSd2 and QRSd1 (ΔQRSd) was shorter in Group 1 (45.67 [22.79] ms) than in Group 2 (49.63 [23.76] ms), although the difference was not statistically significant (*P* = 0.428) (Figure 1). The mean [SD] preoperative PR interval was similar between the two groups (Group 1:127 [18.0] ms *vs.* Group 2:128 [26.0] ms, *P* = 0.870) (Table 2). At follow-up, there was no difference in the mean [SD] PR interval between the two groups (Group 1:133 [24.0] ms *vs.* Group 2:133 [21.0] ms, *P* = 0.950).

### RV dilatation and ΔQRSd association

The association of RV dilatation with ΔQRSd, the correlation between the TR and RVOT pressure gradient, and ΔQRSd were analyzed (Table 3). 24 patients in Group 1 had no RV dilatation compared to only 10 patients in Group 2. However, there was no statistical association between the degree of RV dilatation and ΔQRSd (*P* = 0.546). Six patients in Group 1 had mild dilatation (70.67 ms) compared to 15 patients in Group 2 (53.07 ms). There was no difference in the TR gradient for all pediatric patients in both groups before and after surgery. Consistent with this, the RVOT gradients were also similar in both groups.

## Discussion

Our study monitored ECG changes after TOF correction in pediatric patients using the two currently used techniques, VSA and TAP. The main findings suggest that these two techniques are comparable. Postoperative ECG changes following TOF surgical repair showed that QRSd increased remarkably after surgery, regardless of the surgical approach. This could be due to the right bundle branch block (RBBB) induced by the closure of the VSD in both techniques^14^. Usually, RBBB at various levels causes conduction delay to begin immediately after surgery^14,15^.

**Table 3:** Association of RV dilatation with Δ QRSd and the correlation between the TR gradient and RVOT pressure gradient with ΔQRSd.

**ΔQRSd** (**QRSd2–QRSd1)**	**Group 1** **VSA (*n* = 30)**			**Group 2** **TAP (*n* = 30)**		
	***n***	**Mean (SD)**	***P*** ^*^	**n**	**Mean (SD)**	***P*** ^*^
**RV dilatation** No dilatation Mild dilatation Moderate dilatation Severe dilatation	24 6 ——	66.42 (8.32) 70.67 (8.07) ——	0.533	10 15 4 1	50.56 (29.87) 53.07 (15.49) 35.0 (38.24) 34.0	0.546
**TR gradient (mmHg) – Spearman’s rho**	30	−0.347	0.065	30	−0.057	0.769
**RVOT gradient (mmHg) – Spearman’s rho**	30	−0.145	0.445	30	−0.171	0.367

**Notes.**

* Kruskal–Wallis test (Non-parametric approach).

The present study reported a lower ΔQRSd in Group 1 than in Group 2, although the difference was not statistically significant. TOF repair frequently results in electromechanical desynchrony, which presents mechanically as a right-sided septal/apical flash and electronically as a broad QRS duration (QRSd)^11,13,16^. Ineffective RV mechanics are a result of early septal activation, pre-stretching of the RV basal lateral wall, and subsequent post-systolic shortening^13^.

The impact of QRSd is an important determinant of life-threatening arrhythmias in corrected TOF patients^17^. A prolonged QRS complex is linked to an increased risk of sudden cardiac death and ventricular arrhythmia in corrected-TOF patients^18,18,20,21^. It is also closely associated with RV anomalies and is a predictor of malignant ventricular arrhythmias^19^.

Autopsies of TOF patients who died from sudden cardiac death revealed that the RV myocardium had considerable fibrosis at the ventriculotomy site and the septum, with intact conduction tissues^19^.

Children and adults with Fallot are reportedly more prone to ventricular arrhythmias and abrupt death when the QRSd is 170 ms or longer and 180 ms or longer, respectively^20^. Studies have also identified QRSd >120 ms as a risk factor for death in heart failure with preserved systolic function (HFPSF)^22^. In another study, a longer QRS length of 120–149 ms showed higher mortality at 60 months (*P* = 0.001), wherein the rise in QRS prolongation levels was linked to systolic dysfunction documenting graded increases in mortality^23^.

Severe prolongation of QRSd might be related to chronic volume overload of the RV or the presence of chronic pressure causing PV regurgitation. This was reported in patients undergoing TAP post-TOF repair with significant PV regurgitation during follow-up, who had significant residual valvular stenosis^20^.

In the current study, 24 patients in Group 1 had no RV dilatation compared to only 10 patients in Group 2. Studies have shown that TAP exposes patients to chronic PV regurgitation, while VSA might partly relieve pulmonary obstruction, preserve RV function, and lower the frequency of late arrhythmias, all of which are determinants of long-term outcomes^6,24^. Patients with congenital pulmonary stenosis or corrected TOF frequently experience PV regurgitation, which poses a serious threat, such as arrhythmias and an array of cardiac disorders^25^.

Recently, research has revealed that prolonged PR intervals may be linked to higher mortality and morbidity^26–28^. Atrial fibrillation and pacemaker implantation are both more likely to occur when the PR interval is prolonged^26,27^. In a community-based study, prolonged PR interval was directly related to all-cause mortality^28^. Moreover, late prolongation of the PR interval, which is synonymous with atrioventricular conduction on standard 12-lead ECG, is also clinically important when considering the risk of developing lethal arrhythmias in patients after TOF repair, along with patients suffering from ischemic heart disease or heart failure^29^. In the present study, the PR interval was similar between the two groups in both the baseline ECG and the last follow-up, indicating no significant changes post-TOF repair.

## Limitations

This study has several limitations. First, the sample size of 60 patients is relatively small, which may impact its power in detecting significant differences between the two groups. Although a larger multicenter study would provide more comprehensive insights, such a study may not be feasible in the near future. Second, the follow-up duration of approximately three years is relatively short and may not fully capture the long-term effects of surgical techniques on QRS duration prolongation and associated outcomes. Other studies have reported follow-up periods of 20–30 years^7,8,24^, which are more suitable for assessing long-term impacts. Additionally, the QRS duration in our study groups was not prolonged to the extent associated with possible adverse events^18–21^, suggesting that QRS prolongation might require more time to develop and could be linked to the degree of right ventricular dilatation, regardless of the surgical technique used in TOF repair. Future studies with larger sample sizes and longer follow-up periods are needed to effectively evaluate the association between the surgical approach, QRS prolongation, and the risk of sudden cardiac death. Additionally, although subjects in both study groups underwent ventriculotomy, which may minimize the impact of this confounding factor, our study did not include a valve-sparing approach via exclusive transatrial access. This approach may theoretically have a lesser effect on the QRS duration, and future studies are needed to evaluate this potential benefit. Lastly, the retrospective nature of this analysis, which was conducted using recorded data from a single center with a small cohort of pediatric patients, may also limit the generalizability of our findings.

## Conclusion

This study compared the outcomes of the pulmonary valve-sparing approach (VSA) and transannular patch (TAP) in patients undergoing tetralogy of Fallot (TOF) repair, focusing on changes in QRS duration (QRSd) during short-term follow-up. Both the surgical techniques demonstrated similar postoperative QRSd outcomes. However, the short follow-up duration limits definitive conclusions regarding the long-term effects. Long-term studies are essential to elucidate the durability of QRSd changes over time and their implications for clinical outcomes in patients with TOF undergoing surgical interventions.

## Acknowledgement

Editorial support provided by Dr. Priyanka Biswas Karmakar and Ms. Shaswati Das of Turacoz Healthcare Solutions are appreciated.

## Conflicts of interest

The authors report no conflict of interest.

## Funding

No related funding sources to disclose.
